# X-radiation inhibits histone deacetylase 1 and 2, upregulates Axin expression and induces apoptosis in non-small cell lung cancer

**DOI:** 10.1186/1748-717X-7-183

**Published:** 2012-10-31

**Authors:** Yang Han, Yong Zhang, Lian-he Yang, Xiao-yi Mi, Shun-dong Dai, Qing-chang Li, Hong-tao Xu, Juan-han Yu, Guang Li, Jing Zhao, Chong Han, Xi-ming Yuan, En-hua Wang

**Affiliations:** 1 Department of Pathology, College of Basic Medical Sciences, First Affiliated Hospital of China Medical University, North 2nd Road 92, Heping Ward, Shenyang, 110001, People’s Republic of China; 2 Department of Radiation Oncology, First Affiliated Hospital of China Medical University, Shenyang, People’s Republic of China; 3 Experimental Pathology, Faculty of Health Sciences, Linköping University, Linköping, Sweden

**Keywords:** HDAC1, HDAC2, Axin, Lung neoplasm, Radiosensitization

## Abstract

**Background:**

Histone deacetylase (HDAC) plays an important role in the deacetylation of histone, which can alter gene expression patterns and affect cell behavior associated with malignant transformation. The aims of this study were to investigate the relationships between HDAC1, HDAC2, clinicopathologic characteristics, patient prognosis and apoptosis, to clarify the mechanism of upregulation of the Axis inhibitor Axin (an important regulator of the Wnt pathway) by X-radiation and to elucidate the effect of siRNA on radiation therapy of non-small cell lung cancer (NSCLC).

**Methods:**

HDAC1 and HDAC2 expression levels were measured by immunohistochemistry and reverse transcription PCR. Apoptosis was determined by terminal deoxynucleotidyl transferase-mediated dUTP-nick end labeling and fluorescence activated cell sorting. BE1 cells expressing Axin were exposed to 2 Gy of X-radiation.

**Results:**

Expression of HDAC1 and that of HDAC2 were correlated, and significantly higher in NSCLC tissues than in normal lung tissues (*P* < 0.05). HDAC1 and HDAC2 expression was correlated with pTNM stage and negatively correlated with differentiation of NSCLC and apoptotic index (*P* < 0.05). The prognosis of patients with low expression of HDAC1 and HDAC2 was better than that of those with high expression. X-radiation and siRNA inhibited HDAC1 and HDAC2 expression in NSCLC cells and Axin levels were significantly higher in BE1 cells.

**Conclusions:**

X-radiation and siRNA inhibit expression of HDAC1 and HDAC2, weaken the inhibitory effect of HDAC on Axin, upregulate Axin expression and induce apoptosis of lung cancer cells. Inhibition of HDAC1 and HDAC2 is a means of enhancing the radiosensitivity of NSCLC.

## Background

The Axis inhibitor (Axin) is the most important negative regulatory factor in the Wnt signaling pathway, forming the adenomatous polyposis coli (APC) multiprotein complex with APC and glycogen synthase kinase-3β (GSK-3β) to promote the degradation of β-catenin and block Wnt signaling [1-3]. Our previous study found that Axin can induce apoptosis and enhance the effect of X-radiation in the treatment of non-small cell lung cancer (NSCLC). X-radiation can upregulate Axin expression in certain NSCLC tissues and induce apoptosis of NSCLC cells by p53 and/or the JNK pathway [4]. Why Axin in other NSCLC tissues is not upregulated by X-radiation, and the mechanism of upregulation, remain unclear.

Recent studies show that histone deacetylase (HDAC) plays an important role in the deacetylation of histone, which can alter gene expression patterns and affect cell behavior associated with malignant transformation [5-8]. We suggest that HDAC might regulate the expression of Axin in NSCLCs exposed to X-radiation. In this study, we aimed investigate the relationships between HDAC1, HDAC2, clinicopathologic characteristics, patient prognosis and apoptosis, to clarify the mechanism of Axin upregulation by X-radiation and to elucidate the effect of siRNA on radiation therapy of NSCLC.

## Methods

### Patients

NSCLC tissues (95 cases) and normal lung tissues (20 cases) were collected during surgical procedures performed in the First Affiliated Hospital of China Medical University. Patient survival time was measured as the interval between the date of surgery and the date of death due to recurrence/metastasis or the last follow-up visit. There were 72 males and 23 females, with a median age of 58 years (33–76 years). According to the TNM classification of the International Union Against Cancer (2010), our samples comprised 40 stage I, 18 stage II, 35 stage III and two stage IV tumors. According to pathologic type, there were 45 squamous carcinomas, 40 adenocarcinomas and 10 others. Twenty-nine tumors were well differentiated, 24 moderately differentiated and 42 poorly differentiated.

The study was conducted under the regulations of the Institutional Review Board of China Medical University. Informed consent was obtained from all enrolled patients before surgery.

### Cell culture, vectors, transfection and siRNAs

BE1 cells (a type of lung large cell carcinoma) were kindly provided by Dr. Jie Zheng of the Medical School, Peking University. pFLAG-CMV-5b-Axin was kindly provided by Prof. Mark A. Perrella of Brigham & Women’s Hospital, Boston, MA as described previously. pFlag-CMV-5b was purchased from Sigma-Aldrich (St. Louis, MO). Cells were cultured in Dulbecco’s modified Eagle’s medium (Gibco-BRL, Gaithersburg, MD) with 10% fetal calf serum. Untreated controls received vehicle alone. Eighty percent confluent cells were transfected with pFlag-CMV-5b-Axin using Lipofectamine-2000 (Invitrogen, Carlsbad, CA, USA) according to the manufacturer’s instructions. Double stranded siRNAs were transfected at a final concentration of 50 nM using oligofectamine (Invitrogen, Karlsruhe, Germany) according to the manufacturer’s protocol. siRNAs were purchased from TaKaRa (Dalian, China). The sequences of the siRNAs were: control, 5′-C A G T C G C G T T T G C G A C T G G dtdt-3′; HDAC1, 5′-G C A G A T G C A G A G A T T C A A C dtdt-3′; and HDAC2, 5′-G C C T C A T A G A A T C C G C A TG dtdt-3′.

### X-radiation exposure of cell lines

Eighty percent confluent cells were irradiated (2 Gy) with X-radiation (6 MV) and then cultured for 48 h.

### Immunohistochemical staining

Immunohistochemical staining was performed on 4 μm-thick sequential tissue sections using a streptavidin–peroxidase system (Ultrasensitive™; MaiXin, Fuzhou, China) according to the manufacturer’s instructions. Briefly, sections were deparaffinized in xylems and rehydrated with graded alcohols. Each specimen was incubated with 3% hydrogen peroxide, followed by incubation at 4°C overnight with the primary antibodies anti-HDAC1 (1:100; Santa Cruz Biotechnology Inc., Santa Cruz, CA, USA) and anti-HDAC2 (1:100; Santa Cruz Biotechnology Inc.). Biotinylated IgG was used as a secondary antibody (Cell Signaling Technologies, Beverly, MA, USA). Slides were washed with phosphate buffered saline and the antibody reaction was visualized using a fresh substrate solution containing diaminobenzidine. Sections were counterstained with hematoxylin. Immunostained slides were reviewed by two pathologists independently. The proportions of cells exhibiting HDAC1 or HDAC2 expression were categorized as follows: 0, less than 25%; 1, 26–50%; 2, 51–75%; or 3, more than 75%. The staining intensity was categorized by relative intensity as follows: 1, weak; 2, intermediate; or 3, strong. The proportions and intensity scores were then multiplied to obtain a total score. For the final statistical analysis, scores less than 1 were considered negative and scores of 2 or more were considered positive.

### Reverse transcription (RT)-PCR

Total RNA was extracted from tissues or cells using TRIzol reagent (Invitrogen) according to the manufacturer’s instructions. RT-PCR analysis was performed using a TaKaRa RNA PCR Kit (AMV) Ver.3.0 (TaKaRa). Primer sequences were as follows: HDAC1, sense 5′-A G C C A A G A G A G T C A A A A C A G A-3′, antisense 5′-G G T C C A T T C A G G C C A A C T-3′; HDAC2, sense 5′-A G C A T C A G G A T T C T G T T A C G T T A A T G A-3′, antisense 5′-C A A C A C C A T C A C C A T G A T G A A T A T C T-3′; Axin, sense 5′-A C C G A A A G T A C A T T C T T G A T A A C-3′, antisense 5′-T C C A T A C C T G A A C T C T C T G C-3′; and -actin, sense 5′-A G A G C T A C G A G C T G C C T G A C-3′, antisense 5′-A G T A C T T G C G C T C A G G A G G A-3′. PCR conditions were: 94°C for 4 min; 94°C for 1 min, 53°C (HDAC1); 94°C for 4 min; 94°C for 1 min, 52°C (HDAC2); 94°C for 4 min; 94°C for 1 min, 52°C (Axin) and 55°C (β-actin) for 30 s, 72°C for 30 s, 35 cycles; 72°C for 10 min. Products were resolved in 1% agarose gels and bands were visualized by ethidium bromide staining. Densitometric analysis of bands was performed using a BioImaging System (UVP, CA, USA).

### Western blot analysis

Lysate (50 μg) from BE1 cells was resolved by 12% SDS-PAGE and transferred to PVDF membranes (Sigma). Membranes were blocked with 3% fetal bovine serum, incubated with the primary antibodies anti-HDAC1 (1:1000, Santa Cruz Biotechnology Inc.), anti-HDAC2 (1:1000; Santa Cruz Biotechnology Inc.), anti-Axin (1:400; Santa Cruz Biotechnology Inc.) and anti-β-actin (1:2000; A2066; Sigma) overnight at 4°C. Samples were then incubated with horseradish peroxidase-IgG secondary antibody and bands visualized by enhanced chemiluminescence (Thermo Fisher Scientific Inc., Fremont, CA, USA). Band quantification was performed using Quantity One (Bio-Rad, Hercules, CA, USA). Experiments were performed in triplicate.

### Apoptosis assay

Apoptosis in tissues was detected by terminal deoxynucleotidyl transferase-mediated dUTP-nick end labeling (TUNEL) using an *In Situ* Cell Death Detection Kit (Roche Diagnostics GmbH, Mannheim, Germany) with fluorescein, according to manufacturer’s protocol. Apoptosis of BE1 cells was assayed by annexin-V and propidium iodide (PI) staining, detected by flow cytometry (FAC-S Calibur; BD Bioscience, San Jose, CA, USA) and analyzed using Modfit LT V3.0 software (Verity Software House Inc., Topsham, ME, USA). Only annexin-V positive and PI negative cells were considered apoptotic. All experiments were performed in triplicate.

### Statistical analysis

All values were expressed as the mean ± SD. The statistics package SPSS 11.0 (SPSS Inc., Chicago, IL) was used for all analyses. The relationship between percentages of HDAC1 and HDAC2 positive cells and apoptotic index was analyzed by the bivariate correlation analytical method (Spearman’s correlation analysis). Relationships of HDAC1 and HDAC2 with clinical and pathologic factors were analyzed using Pearson’s *χ*^2^ test. Variance analysis (least-significant difference method) was used for the detection of apoptosis on flow cytometry. Survival analysis was performed on the relationship of HDAC1 and HDAC2 with patient prognosis using the Kaplan–Meier method (log-rank test). *P* < 0.05 was considered statistically significant.

## Results

### Correlation of HDAC1 and HDAC2 expression with clinicopathologic factors and apoptosis in NSCLC

**Figure 1:**
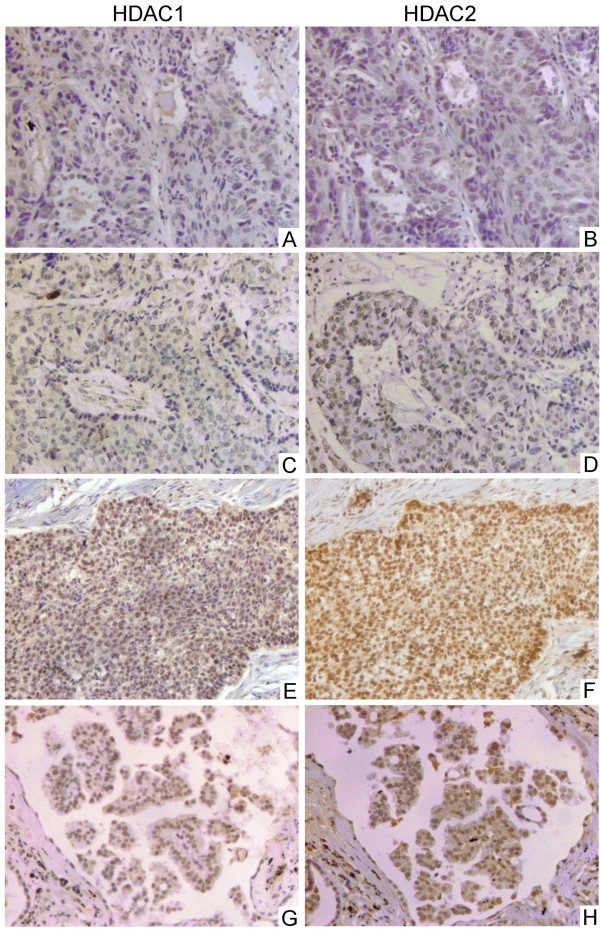
**Correlation between HDAC1 and HDAC2 in NSCLC tissue.** Both HDAC1 and HDAC2 expression is localized to the nucleus. Case 1 (**A**, **B**), squamous cell carcinoma, T_1_N_0_M_0_. Case 2 (**C**, **D**), squamous cell carcinoma, T_2_N_0_M_0_. Case 3 (**E**, **F**), adenocarcinoma, T_3_N_1_M_0_. Case 4 (**G**, **H**), adenocarcinoma, T_1_N_0_M_0_. Immunohistochemical streptavidin–peroxidase method, magnification × 200.

**Figure 2:**
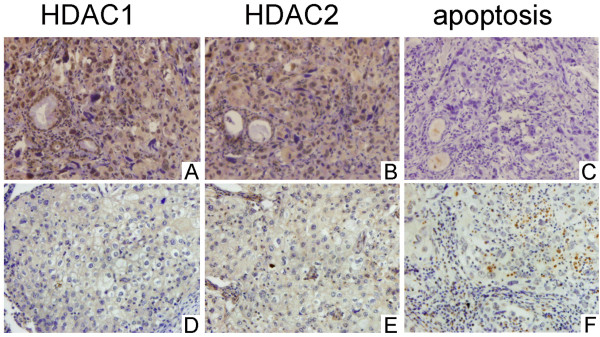
**Correlation between HDAC1 and HDAC2 and apoptosis in NSCLC tissues.** Case 1 (**A**–**C**), positive for expression of HDAC1 and HDAC2 but low apoptotic index. Case 2 (**D**–**F**), negative for expression of HDAC1 and HDAC2 and high apoptotic index. Immunohistochemical streptavidin–peroxidase method and TUNEL, magnification × 200.

Normal lung tissues expressed higher levels of HDAC1 and HDAC2 than NSCLC tissues (positive rates of HDAC1 and HDAC2 in normal lung tissues are both 85.0%, 17/20, positive rate of HDAC1 in NSCLC tissues is 50.5%, 48/95 and positive rate of HDAC2 in NSCLC tissues is 48.4%, 46/95). Expression of HDAC1 appeared to be correlated with expression of HDAC2 (*P* < 0.05). In addition, HDAC1 and HDAC2 expression appeared to be correlated with pTNM stage and negatively correlated with degree of differentiation. The positive rates for well and moderately differentiated cells (37.7%, 20/53 and 37.7%, 20/53, respectively) were significantly lower than those for poorly differentiated cells (66.7%, 28/42 and 61.9%, 26/42, respectively) (*P* < 0.05). The positive rates for stage I-II tumors (41.4%, 24/58 and 39.7%, 23/58, respectively) were significantly lower than those for stage III-IV (64.9%, 24/37 and 62.2%, 23/37, respectively) (Figure 1, Table 1). No significant difference was found in gender, age, histologic type or lymphatic metastasis. TUNEL was used to detect apoptosis in 95 NSCLC tissue samples. HDAC1 and HDAC2 expression were negatively correlated with apoptotic index (rP = –0.315, *P* = 0.002 and rP = –0.322, *P* = 0.002, respectively) (Figure 2).

**Table 1:** Relationships among HDAC1 and HDAC2 in NSCLC tissue and clinicopathologic factors

**Clinicopathologic factor**				**HDAC1 expression**		***P *value**	**HDAC2 expression**		***P *value**
	**–**	**+**		**–**	**+**			
Gender						
Male	38	34	0.254^†^	39	33	
Female	9	14	0.532^†^	10	13	0.372^†^
Age (years)						
< 58	26	21		28	19	
≥ 58	21	27	0.532^†^	21	27	0.123^†^
Histologic type						
SCC	22	23		25	20	
AC	22	18		21	19	
Others	3	7	0.366^†^	3	7	0.339^†^
Differentiation						
Well/Moderate	33	20		33	20	
Poor	14	28	0.005^†^	16	26	0.019^†^
pTNM stage^*^						
I/II	34	24		35	23	
III/IV	13	24	0.026^†^	14	23	0.032^†^
Lymphatic metastasis						
No	27	21		28	20	
Yes	20	27	0.182^†^	21	26	0.183^†^

SCC, squamous cell carcinoma; AC, adenocarcinoma.

^*^TNM staging system of the International Union Against Cancer (2010).

^†^Pearson’s *χ*^2^ test.

### Correlation of HDAC1, HDAC2, apoptotic index and patient prognosis in NSCLC

**Figure 3:**
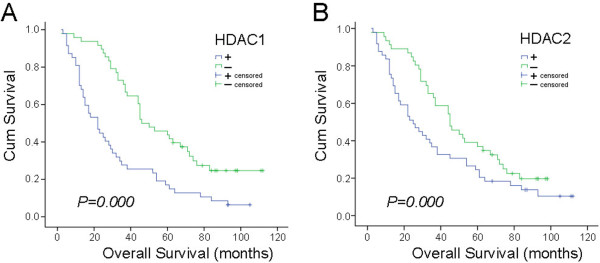
**Relationship between HDAC1 and HDAC2 expression and survival time in NSCLC patients.** HDAC1 and HDAC2 levels and survival from the time of surgery were measured in 95 NSCLC patients. The prognosis of patients negative for HDAC1 and HDAC2 was significantly better than that of HDAC1 and HDAC2 positive patients (*P* = 0.000).

Survival time post-surgery ranged from 2 to 112 months for the 95 cases of NSCLC; the average was 46.7 ± 3.6 months and the median was 36.0 ± 5.2 months. The Kaplan–Meier method (log-rank test) was used to analyze life span. The average survival time of the group negative for HDAC1 expression was 60.6 ± 4.9 months, with a median of 46.0 ± 6.9 months. The average survival time of the group positive for HDAC1 expression was 32.4 ± 4.2 months, with a median of 22.0 ± 4.1 months. The average survival time of the group negative for HDAC2 expression was 52.5 ± 4.3 months, with a median of 45.0 ± 6.3 months. The average survival time of the group positive for HDAC2 expression was 38.7 ± 4.9 months, with a median of 25.0 ± 5.0 months. Statistical analysis indicated that the prognosis of patients who were negative for HDAC1 expression was significantly better than that of those who were positive (*P* = 0.000). The prognosis of patients who were negative for HDAC2 was significantly better than that of those who were positive (*P* = 0.000) (Figure 3).

### X-radiation downregulated expression of HDAC1 and HDAC2 and upregulated Axin expression

**Figure 4:**
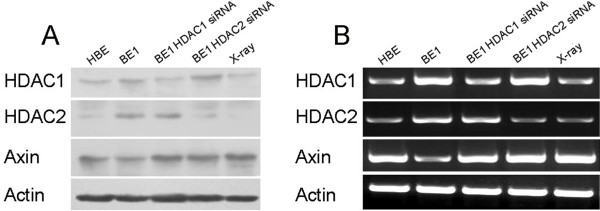
**HDAC1, HDAC2 and Axin expression in lung carcinoma cell line after siRNA and X-radiation exposure.** (**A**) Western blot analysis of lung carcinoma (BE1) cells after siRNA and X-radiation exposure. HDAC1 siRNA inhibited expression of HDAC1, HDAC2 siRNA inhibited expression of HDAC2 and X-radiation inhibited expression of HDAC1 and HDAC2. Both HDAC siRNA and X-radiation upregulated protein expression of Axin. (**B**) RT-PCR analysis of BE1 cells after siRNA and X-radiation exposure. HDAC1 siRNA inhibited expression of HDAC1, HDAC2 siRNA inhibited expression of HDAC2 and X-radiation inhibited expression of HDAC1 and HDAC2. Both HDAC siRNA and X-radiation upregulated RNA expression of Axin. HBE, human bronchial epithelial cells.

To investigate the effect of X-radiation on the expression of HDAC1, HDAC2 and Axin, BE1 cells were exposed to X-radiation (2 Gy) and HDAC1, HDAC2 and Axin expression was measured 24 h later by RT-PCR and Western blot. Expression of HDAC1 and HDAC2 was downregulated, whereas that of Axin was upregulated (Figure 4).

### siRNAs downregulated expression of HDAC1 and HDAC2 and upregulated Axin expression

To investigate the effects of HDAC1 and HDAC2 on the expression of Axin, we used a plasmid-based RNAi method to knock down HDAC1 and HDAC2 expression. Target sequences of human HDAC1 and HDAC2 were designed and ligated into BE1 cells. The recombinant plasmids were screened by BamHI and HindIII digestion and further verified by sequencing. The recombinant and its control plasmid were transfected into cultured BE1 cells. Twenty-four hours later, RNA expression of HDAC1 and HDAC2 was determined by RT-PCR. The RNA levels of HDAC1 and HDAC2 in BE1 cells transfected with pAVU6-siHDAC1 or pAVU6-siHDAC2 were significantly decreased compared with those of cells transfected with an empty vector (EC109-U6). Protein levels of HDAC1, HDAC2 and Axin were measured by Western blot, with results similar to those for their RNA levels (Figure 4). These results show that HDAC1and HDAC2 proteins were efficiently knocked down by siRNA in BE1 cells, whereas Axin protein was upregulated.

### HDAC1 and HDAC2 siRNAs and X-radiation enhanced apoptosis of BE1 cells

**Figure 5:**
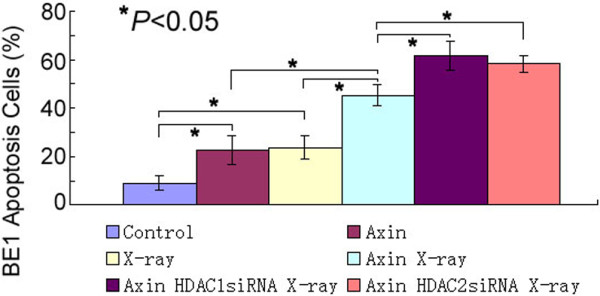
**Effects of Axin, X-radiation and siRNA on apoptosis of BE1 cells.** BE1 cells were transiently transfected with Axin, pretreated with vehicle and exposed or not exposed to X-radiation and HDAC1 and HDAC2 siRNA (measured by flow cytometry). Both X-radiation and HDAC1 and HDAC2 siRNA induced apoptosis of the BE1 cells.

Apoptosis was measured by flow cytometry. Only annexin-V positive and PI negative cells were considered apoptotic. It was found that BE1 cells expressing pFLAG-cmv-5b-Axin exhibited increased apoptosis, to the level of cells exposed to X-radiation. Treatment of the cells with X-radiation further increased apoptosis (45.2 ± 4.1%). In addition, treatment of the cells with HDAC1 or HDAC2 siRNA further increased apoptosis (61.4 ± 5.8% and 58.3 ± 3.4%, respectively). Thus, HDAC1and HDAC2 proteins were efficiently knocked down by siRNAs in BE1 cells, whereas Axin protein was upregulated and apoptosis increased (Figure 5, Additional file 1).

## Discussion

NSCLC accounts for about 80% of cases of lung cancer [9]; however, the use and therapeutic effects of radiation therapy are limited by the hyposensitivity of NSCLC to radiation [10-13]. Our previous study found that Axin can induce apoptosis and enhance the effects of X-radiation in some cases of NSCLC, by upregulating Axin expression in certain parts of the NSCLC and inducing apoptosis of NSCLC cells by p53 and/or the JNK pathway [4]. It remains unclear why Axin is not upregulated by X-radiation in other parts of NSCLCs. In these regions, apoptosis is not upregulated, leading to failure of radiotherapy. The mechanism by which X-radiation upregulates Axin expression is also unclear.

The acetylation level of histone and non-histone proteins in the nucleolus determines chromatin structure. High levels of histone acetylation lead to an open chromatin configuration, providing access for specific transcription factors and the general transcription machinery [14,15]. HDACs deacetylate the ε-amino group of lysines located at the N-terminal tail of histones, leading to a tightly packed chromatin formation (heterochromatin) and the suppression of gene expression [5,16,17]. Based on the fact that in some NSCLCs Axin is not upregulated by X-radiation [4], we hypothesized that HDAC1 and HDAC2 may be inhibitors of the upregulation of Axin by X-radiation, and designed experiments to test this. First, we measured protein expression of HDAC1 and HDAC2 in 95 NSCLC tissue samples by immunohistochemistry, and found that it was related to pTNM stage and negatively correlated with the degree of differentiation of NSCLC. These results indicate that HDAC1 and HDAC2 can play important roles in the pathologic diagnosis and may be targets for knock-down in treatment of NSCLC. Our previous study found that Axin expression is positively correlated with apoptotic index, and the prognosis of patients positive for Axin was significantly better than that of Axin-negative patients.

Given our findings that the prognosis of patients negative for HDAC1 and HDAC2 was significantly better than that those who were positive, and that HDAC2 expression is negatively correlated with apoptotic index, we designed siRNAs to knock down HDAC1 and HDAC2 and measured Axin expression and apoptosis in BE1 cells (a type of large cell lung carcinoma). We found both RNA and protein expression of Axin to be upregulated after HDAC1 and HDAC2 had been knocked down by siRNA. These results suggest that Axin may be related to HDAC1 and HDAC2 levels in NSCLC tissues and cells, and that HDAC1 and HDAC2 can inhibit Axin expression.

The hyposensitivity of NSCLC to X-radiation is a major reason for failure of radiotherapy. Our previous study showed that transfection of Axin into NSCLC cells enhances their radiosensitivity [4]. X-radiation increases Axin expression and induces apoptosis in NSCLC tissues, though the mechanism remains unclear. In the present study, we found that both siRNA and X-radiation can downregulate the expression of HDAC1 and HDAC2. Combining our previous results showing that X-radiation upregulates Axin expression in some NSCLC tissues, we suggest that X-radiation may inhibit HDAC1 and HDAC2 expression and weaken the inhibitory effect of HDAC1 and HDAC2 on Axin, thereby upregulating Axin expression and inducing apoptosis of cancer cells. Our finding that, in some, NSCLCs, Axin was not upregulated by X-radiation [4] is explained by our observation in the present study that X-radiation did not completely inhibit HDAC1 and HDAC2 in NSCLC tissues and cells. Flow cytometry showed that siRNAs of HDAC1 and HDAC2 induced apoptosis of BE1 cells and enhanced the apoptosis-inducing effect of X-radiation.

Aberrant expression of HDACs has been found in various types of cancer. For example, expression of HDAC1 mRNA in human urinary bladder cancer specimens was significantly higher than that in normal controls [18], and increased mRNA expression of HDCA1 and HDAC2 was detected in ovarian cancer tissues compared with normal tissue samples [19]. Moreover, expression of HDAC1 mRNA was associated with clinicopathologic factors such as tumor size and histologic grade in human breast cancer, gastric cancer and glioblastoma [20-22]. These findings suggest that HDACs might be targets for cancer therapy [23-25].

## Conclusions

We believe this study not only clarifies the issue of why X-radiation upregulates Axin and induces apoptosis, and explains why in some NSCLCs Axin is not upregulated by X-radiation, but also elucidates the effect of siRNA on radiation therapy of NSCLC.

## Competing interests

The authors declare that there is no competing interests.

## Authors’ contributions

YH participated in the design of the study, performed data collection, statistical analysis and drafted the manuscript. YZ, LY, XM, SD and QL participated in the design and coordination of the study and data collection. YH, LY, HX, JY and CH carried out the immunoassays and protein analyses. GL, JZ, XY and EW participated in the design of the study and interpretation of the data, EW also assisted with the statistical analysis. All authors read and approved the final manuscript.

## Funding

This study was supported by grants from the National Natural Science Foundation of China (No.81000995 to Y. Han).

## Supplementary Material

Additional file 1 The apoptosis of the BE1 cells, measured by flow cytometry. Click here for file
