# Supplementary figures and images for: X-radiation inhibits histone deacetylase 1 and 2, upregulates Axin expression and induces apoptosis in non-small cell lung cancer
Source: Radiat Oncol. 2012 Oct 31;7:183. doi: 10.1186/1748-717X-7-183 (PMC3542190; doi:10.1186/1748-717X-7-183)

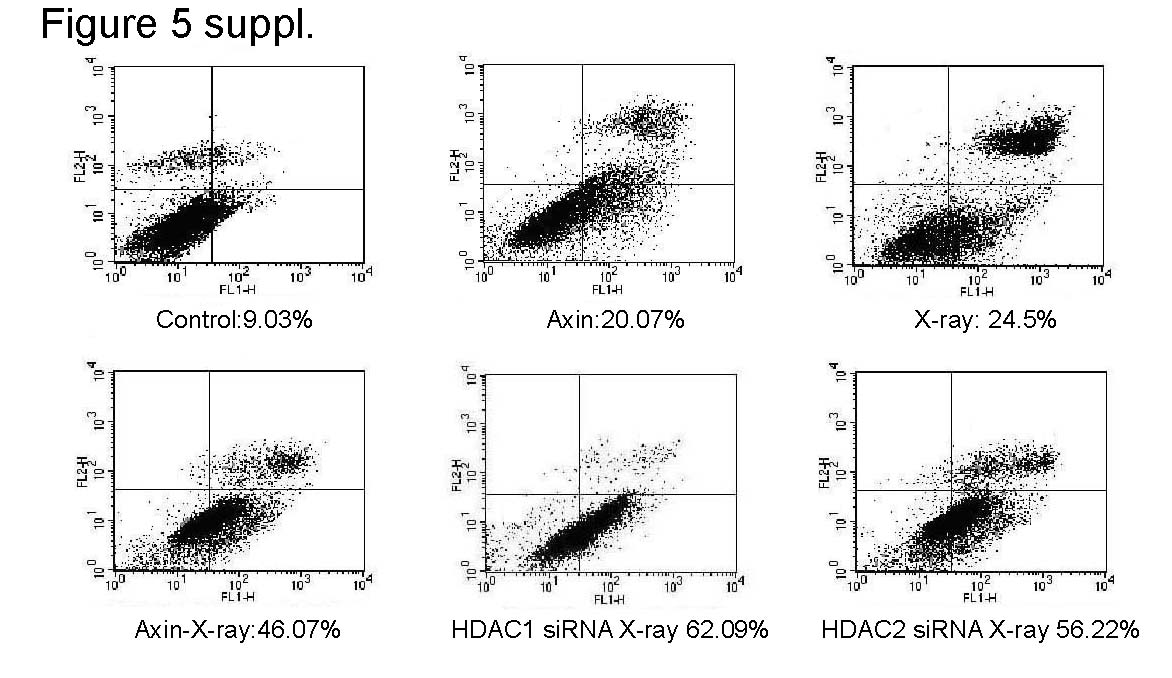

Supplement: Additional file 1 — The apoptosis of the BE1 cells, measured by flow cytometry. [file 1748-717X-7-183-S1.tiff]
